# Changes in the Proteome of the Circle of Willis during Aging Reveal Signatures of Vascular Disease

**DOI:** 10.1155/2024/4887877

**Published:** 2024-06-26

**Authors:** Vikram Subramanian, Denise Juhr, Lydia S. Johnson, Justin B. Yem, Piero Giansanti, Isabella M. Grumbach

**Affiliations:** ^1^ Abboud Cardiovascular Research Center Department of Internal Medicine Carver College of Medicine University of Iowa, Iowa City, USA; ^2^ Bavarian Center for Biomolecular Mass Spectrometry (BayBioMS@MRI) Technical University of Munich, Munich, Germany; ^3^ Free Radical and Radiation Biology Program Department of Radiation Oncology Carver College of Medicine University of Iowa, Iowa City, USA; ^4^ Iowa City VA Healthcare System, Iowa City, IA, USA

## Abstract

Approximately 70% of all strokes occur in patients over 65 years old, and stroke increases the risk of developing dementia. The circle of Willis (CoW), the ring of arteries at the base of the brain, links the intracerebral arteries to one another to maintain adequate cerebral perfusion. The CoW proteome is affected in cerebrovascular and neurodegenerative diseases, but changes related to aging have not been described. Here, we report on a quantitative proteomics analysis comparing the CoW from five young (2–3-month-old) and five aged male (18–20-month-old) mice using gene ontology (GO) enrichment, ingenuity pathway analysis (IPA), and iPathwayGuide tools. This revealed 242 proteins that were significantly dysregulated with aging, among which 189 were upregulated and 53 downregulated. GO enrichment-based analysis identified blood coagulation as the top biological function that changed with age and integrin binding and extracellular matrix constituents as the top molecular functions. Consistent with these findings, iPathwayGuide-based impact analysis revealed associations between aging and the complement and coagulation, platelet activation, ECM–receptor interaction, and metabolic process pathways. Furthermore, IPA analysis revealed the enrichment of 97 canonical pathways that contribute to inflammatory responses, as well as 59 inflammation-associated upstream regulators including 39 transcription factors and 20 cytokines. Thus, aging-associated changes in the CoW proteome in male mice demonstrate increases in metabolic, thrombotic, and inflammatory processes.

## 1. Introduction

Aging is a complex biological process characterized by numerous physiological and structural changes. Age-related alterations in the cerebral arteries of humans predispose to neurodegenerative diseases and cognitive decline and promote cerebrovascular diseases such as intracerebral hemorrhage, microbleeds, and stroke [1, 2, 3]. While resistance arteries, penetrating and parenchymal arterioles, and cerebral capillaries are the principle sites for age-dependent loss of functionality and disease development, the larger arteries of the mouse circle of Willis (CoW) display reduced cerebral blood flow and elastin deposition with aging, as well as increased arterial stiffness and collagen deposition [4]. In the aged human middle cerebral artery, functional changes in both vasodilation and constriction have been reported [5]. Elucidating changes in the proteome provides insight into the mechanisms that trigger arterial aging and is expected to facilitate the development of treatments for, or the prevention of, cerebrovascular diseases that are linked with old age.

The CoW is a ringlike arterial structure at the base of the brain and ensures collateral blood flow between the posterior and anterior cerebral arterial systems [6]. It consists of the two branches of the internal carotid artery, the vertebrobasilar artery, and the anterior and posterior communicating arteries [7]. Healthy circulation is vital for cerebral perfusion and function and to prevent ischemia-associated damage [8, 9]. Several molecular and physiological studies revealed changes in the structure of the CoW and its surface branches and in protein expression within the CoW in healthy aging [10, 11] and in cerebrovascular and neurodegenerative diseases [10, 12]. Also, studies of brains of aged mice and humans have reported hypoplasia of the vascular wall [13], decreases in cerebral blood flow [14], ischemic stroke [15], changes in the thickness of the arterial wall, and loss of elasticity of the CoW arteries [16]. In animal models, age-induced changes in brain microvessels included disruption of the blood–brain barrier [17] with changes of the basement membrane [18], decreased blood velocity [19], impairment of bioenergetic pathways [20], and increases in oxidative stress [18]. However, information on age-induced impairment specifically in the CoW is scarce. Although recent quantitative analyses of the proteome have contributed to our understanding vascular and neurodegenerative disorders [21, 22], few studies have focused on age-related changes in the cerebral arteries of the CoW [16, 23].

In this study, we performed an unbiased, quantitative analysis of the proteomes in the cerebral arteries of the CoW of young (2–3-month-old) and aged (18−20-month-old) mice. The primary goal was to characterize the impact of aging on expression and relative abundance of proteins in cerebral arteries of young and aged healthy mice; the secondary goal was to identify protein networks and pathways that may cause age-related changes in vascular structure and function and increase the risk of cerebrovascular dysfunction in the elderly population; and the tertiary goal was to identify age-related changes in the proteomes of specifically endothelial and smooth muscle cells of the CoW arteries.

## 2. Materials and Methods

### 2.1. Animals and Housing

All experimental procedures were approved by the Institutional Animal Care and Use Committees of the University of Iowa and the Iowa City VA Health Care System, and they complied with the standards of the Institute for Laboratory Animal Research. To avoid gender-related confounding differences, only male C57BL/6J mice were used. Five mice each were allocated to the young and aged groups, for analysis of the CoW proteome at 2–3 months of age and 18–20 months of age, respectively. Mice were housed in temperature-controlled rooms and maintained on a dark/light cycle of 12 hr, standard rodent chow, and water ad libitum.

### 2.2. Isolation of CoW and Preparation of Protein Samples

Mice were euthanized by inhalation of 100% CO_2_ followed by harvest of the brain according to an institutionally approved euthanasia protocol. After the brain was isolated, the cerebral arteries of the CoW (anterior, posterior and middle) and their main branches were surgically removed from the base of the brain [24]. Thereafter, each isolated CoW was rinsed with cold Dulbecco's phosphate-buffered saline solution (DPBS) (Gibco # 2430024) and stored at −80°C until further use. For protein extraction, the CoW was mixed with 80 *μ*L of RIPA buffer (Fisher Scientific # R0278) containing protease (PierceTM # A32963) and phosphatase inhibitor (PierceTM # A32957) cocktails and transferred to fresh Eppendorf tubes (Corning # 3207). The mixture was vortexed three times for 30 s (Fisher Scientific # 0215370) and gently agitated for 30 min at 15–20°C. Samples were then vortexed and incubated (VWR # 12621-112) at 95°C for 10 min. Samples were spun down and subjected to tissue lysis using a Covaris E220 focused ultrasonicator (Covaris # 500239). For this purpose, samples were transferred to Covaris microfiber Screwcap tubes (Covaris # 520216) and kept on ice during the procedure. The instrument parameters for shearing were as follows: water level set point 10, water temperature 6°C, peak incident power 175 W, duty factor 10%, cycles per burst 200, and duration 300 s. The homogenized lysates were transferred to fresh Eppendorf tubes and centrifuged at 16,100x *g* (Eppendorf # 5415R) for 30 min. The supernatant was collected and stored at −80°C until further use. The total protein concentration was measured using a Bicinchoninic acid (BCA) protein assay kit (PierceTM # 23225). A 30-*µ*g sample of protein from each biological replicate was used for quantitative proteomics analysis.

### 2.3. Protein Digestion

Protein digestion was performed according to a previous publication [25] using an single pot, solid-phase enhanced sample preparation (SP3) method [26]. For each sample, 30 *μ*g of protein in 150 *μ*L lysis buffer was incubated with 10 *μ*L of SP3 beads (1 : 1 mix of Sera-Mag Speed Beads A and B (Thermo Scientific)). Pure acetonitrile (ACN) (VWR Chemicals) was added (directly to the samples) to a final concentration of 70% (v/v). Samples were incubated in a thermal shaker for 18 min at 800 rpm and then transferred to the magnet rack for 3 min to immobilize the SP3 beads. The supernatant was discarded, and the SP3 beads were rinsed three times with 1 mL of 80% (v/v) ethanol/water and once with 800 *μ*L of ACN. The bound proteins were reduced by adding 100 *μ*L of 10 mM 1,4-dithiothreitol (DTT) (Sigma) in 50 mM ammonium bicarbonate (Sigma), pH 8.0, incubated at 37°C with shaking at 800 rpm for 1 hr. Proteins were alkylated with 55 mM 2-chloroacetamide (CAA) (Sigma) for 1 hr at 37°C, in the dark. Finally, 1 *μ*g of trypsin (Thermo Scientific) was added, and samples were incubated overnight at 37°C with shaking at 800 rpm. After protein digestion, samples were acidified with formic acid (FA, Carlo Erba) at a final concentration of 1% (v/v), dried in vacuo, and stored at −80°C until further use.

### 2.4. Automated Off-Line Fractionation

For whole proteome analysis, peptides were resuspended in 110 *μ*L of buffer A (25 mM ammonium formate (Sigma), pH 10), and subjected to high pH reverse phase fractionation using the AssayMAP Bravo platform and 5 mL RP-S cartridges (Agilent). The cartridges were primed sequentially with 150 *μ*L isopropanol (Chemsolute), ACN, and buffer B (80% ACN in 10 mM ammonium formate, pH 10), at a flow rate of 50 *μ*L/min. Subsequentially, cartridges were equilibrated with 100 *μ*L of buffer A, and peptides were then loaded at 5 *μ*L/min. The flow-through (FT) was collected. Peptides were then eluted with 25 mM ammonium formate, pH 10, using increasing concentrations of ACN (5%, 10%, 15%, 20%, 25%, 30%, and 80%). The seven flow-through fractions were pooled into four (5% + 25%, 10% + 30%, 15% + 80%, and 20% + FT), dried in vacuo, and stored at −80°C until further use.

### 2.5. LC-MS/MS Analysis

Nanoflow LC-MS/MS measurements were performed using a Dionex Ultimate 3000 UHPLC + system coupled to an Orbitrap Eclipse mass spectrometer (Thermo Fisher Scientific). Peptides were delivered to a trap column (75 *μ*M i.d. × 2 cm, packed in-house with 5 *μ*m Reprosil C18 beads, Dr. Maisch) and washed using 0.1% FA at a flow rate of 5 *μ*L/min for 10 min. Subsequently, peptides were transferred to an analytical column (75 *μ*m i.d. × 45 cm, packed in-house with 3 *μ*m Reprosil C18 beads, Dr. Maisch) at a flow rate of 300 nL/min. Peptides were chromatographically separated using a linear gradient of solvent B (0.1% FA, 5% DMSO (Sigma) in ACN and solvent A (0.1% FA, 5% DMSO in water). Linear gradients were as follows: from 4% to 32% of B in 80 min, from 32% to 80% B in 2 min, 80% B for further 2 min, and from 80% to 2% B in 2 min. The total measurement time was 90 min. Full-scan MS spectra were recorded in the Orbitrap from 360 to 1,300 m/z at a resolution of 60,000 using an automatic gain control (AGC) target value of 100% and maximum injection time (maxIT) of 50 ms. After the survey scan was completed, the most intense precursors were isolated using an isolation window of 1.3 m/z for high energy collisional dissociation (HCD) fragmentation, and fragment ions were recorded in the Orbitrap at a resolution of 15,000, with a maxIT of 22 ms and AGC of 200%. Normalized collision energy (NCE) was set to 25%. Charge state screening was enabled, and only precursors with charge states between 2 and 6 were selected for fragmentation, within a cycle time of 2 s. Dynamic exclusion was set to 35 s.

### 2.6. Identification and Quantitation of Peptides and Proteins

Raw mass spectrometry data were processed using the MaxQuant software (version 2.2.0.0) with its built-in search engine, Andromeda [27]. Spectra were searched against the UniProtKB database (*Mus musculus*, UP000000589, 55,338 entries downloaded on 10.2022). Enzyme specificity was set to trypsin, allowing for two missed cleavages, and the search included cysteine carbamidomethylation as a fixed modification and protein N-term acetylation and methionine oxidation as variable modifications. Identifications were adjusted to 1% false discovery rate (FDR) at protein and peptide levels. The match-in-between runs and the second peptide options were enabled. The MaxLFQ algorithm [28] was used for label-free quantification (LFQ). The mass spectrometry proteomics data have been deposited in the ProteomeXchange Consortium via the PRIDE partner repository [29] with the dataset identifier PXD043001.

### 2.7. Proteomics Data Analysis

Data analysis was performed using the Perseus software (version 2.0.7.0.). Protein identifications were filtered to remove contaminants and decoy hits before performing data normalization of the log2-transformed LFQ intensity values by median centering, as implemented in Perseus. For statistical analysis, only proteins that had been quantified in at least four biological replicates were retained, and missing values for the fifth replicate were imputed from the normal distribution in Perseus, using default parameters. For all samples, the amount of imputed data was below 10%. *Supplementary 1* lists all measurements (before imputation) and *Supplementary 2* data sets after imputation. Proteins found to be significantly regulated were identified using the Student's *t*-test with an S0 parameter of 0.1 and corrected for multiple hypotheses using a permutation-based FDR of 5%. Significantly dysregulated proteins were defined as those for which at least two unique peptides were present at a ratio of <0.77-fold or >1.30-fold following correction of *p* values according to previous publications [30, 31, 32].

### 2.8. Pathway Enrichment Analysis

The hierarchical cluster analysis of all dysregulated proteins was performed using GraphBio [33] and SR plot, according to a previous publication [34]. A volcano plot of differentially expressed proteins was also constructed using VolcaNoseR [35]. Enrichment analysis of differentially expressed proteins was performed to identify the biological processes, cellular locations, molecular functions, and Kyoto Encyclopedia of Genes and Genomes (KEGG) pathways that they influence. These analyses were done using the HemI 2.0 (https://hemi.biocuckoo.org/) [36], ExpressAnalyst (www.expressanalyst.ca), and Enrichr [37] webtools. The canonical pathway and upstream regulator analyses were performed using the INGENUITY Pathway Analysis (IPA) software (http://www.INGENUITY.com) [38]. For the analysis, we applied a threshold of −log (*p* value) >2. Pathways with a *z*-score of >2.0 were considered activated and those with a score of <–2.0 inhibited. The impact pathway analysis on differentially expressed proteins was executed using iPathwayGuide [39, 40]. iPathwayGuide pathway annotations were obtained from the KEGG database, release 100.0+/11−12 (Nov 21), and gene ontology annotations from the Gene Ontology Consortium database, release 2021 November 4 [41]. The pathways identified were considered to be affected if the FDR-corrected *p* value threshold was <0.05. Analysis of protein functions and protein–protein interactions, as well as functional characterization of dysregulated proteins, was performed by coupling the STRING and Reactome databases (http://string-db.org) [42, 43]. To identify the age-associated changes in the mitochondrial proteome of the CoW, data were mapped to MitoCarta 3.0 database, an inventory of mammalian mitochondrial proteins and pathways [44]. In addition, we performed a comparison of proteomics data from our study and from a previous study [16]. Novel proteins that were identified in our dataset only and all identified proteins from current study and previous study were analyzed using ExpressAnalyst webtool (www.expressanalyst.ca) for pathway enrichment analysis (KEGG pathway) and gene ontology analysis for cellular components. Top enriched pathways, total number of proteins associated to pathway, and their cellular locations in aging CoW were studied. Top 10 enriched KEGG pathways and cellular components from two datasets were selected and compared to each other. Age-associated changes in the endothelial and vascular smooth muscle proteins of the CoW were identified by mapping the significantly dysregulated proteins to an in-house human microvascular endothelial cell proteome dataset (unpublished study) and a published vascular smooth muscle proteome dataset [45].

### 2.9. Statistical Analysis

All experiments were performed in five biological replicates. The data were expressed as mean with standard error, and the statistical analysis was performed using GraphPad Prism 9.0 software. Normal distribution was assessed by D'Agostino–Pearson omnibus normality test. An unpaired Student's *t*-test or the nonparametric Mann–Whitney test was used to determine statistical significance for comparison of the two groups. Differences were considered as significant if the *p* values were <0.05.

## 3. Results

### 3.1. Proteomic Profiling of the Mouse CoW

**Figure 1:**
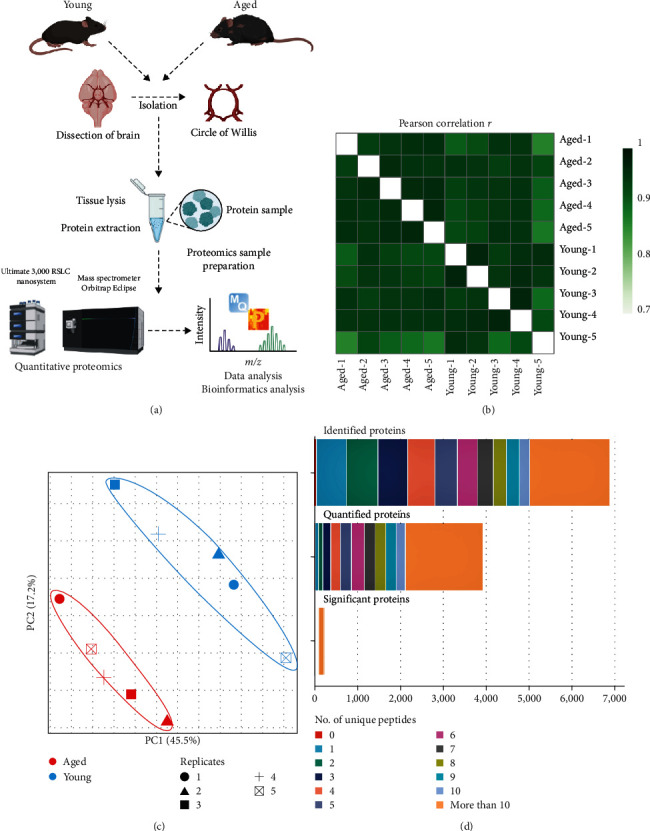
Quantitative proteomics analysis of the CoW. (a) Workflow for high-throughput identification and quantification of proteins from the circle of Willis (CoW) of young and aged mice: CoW isolation, tissue lysis and protein extraction, sample preparation, mass spectrometry, and data analysis. (b) Matrix representation of Pearson correlation values based on label-free quantification (LFQ) intensities, quantifying reproducibility in samples from young and aged mice. (c) Plot of principal component analysis (PCA) outcomes for proteins identified in biological replicates of young and aged mice. (d) Distribution of the number of unique peptides in all identified, quantified, and significantly dysregulated proteins.

To understand age-induced changes in protein expression in CoW arteries, we performed quantitative proteomics using LC-MS/MS on samples from 2–3 months old (young) and 18–20 months old (aged) mice (Figure 1(a)). We identified 6,874 proteins in the two groups, of those, 3,926 proteins were quantified with at least two unique peptides (*Supplementary 1 and 2*). Label-free quantitation (LFQ) of the measured proteins among the replicates was highly reproducible, as assessed by the Pearson correlation (Figure 1(b)). Principal component analysis (PCA) showed that age is a key variable (Figure 1(c)). The distribution of unique peptides among the identified, quantified, and significantly dysregulated proteins is shown in Figure 1(d). A protein was classified as identified if a valid MS/MS spectrum was available for at least one of its peptides: as quantified if the protein was identified in all five biological replicates and as significantly dysregulated if its relative abundance in the two groups differed with statistical significance. Among 3,926 quantified proteins for which at least two unique peptides were available (*Supplementary 3*), 242 proteins had significantly different expression profiles in the aged versus young groups (±1.3-fold; *p*  < 0.05) (Table 1).

**Figure 2:**
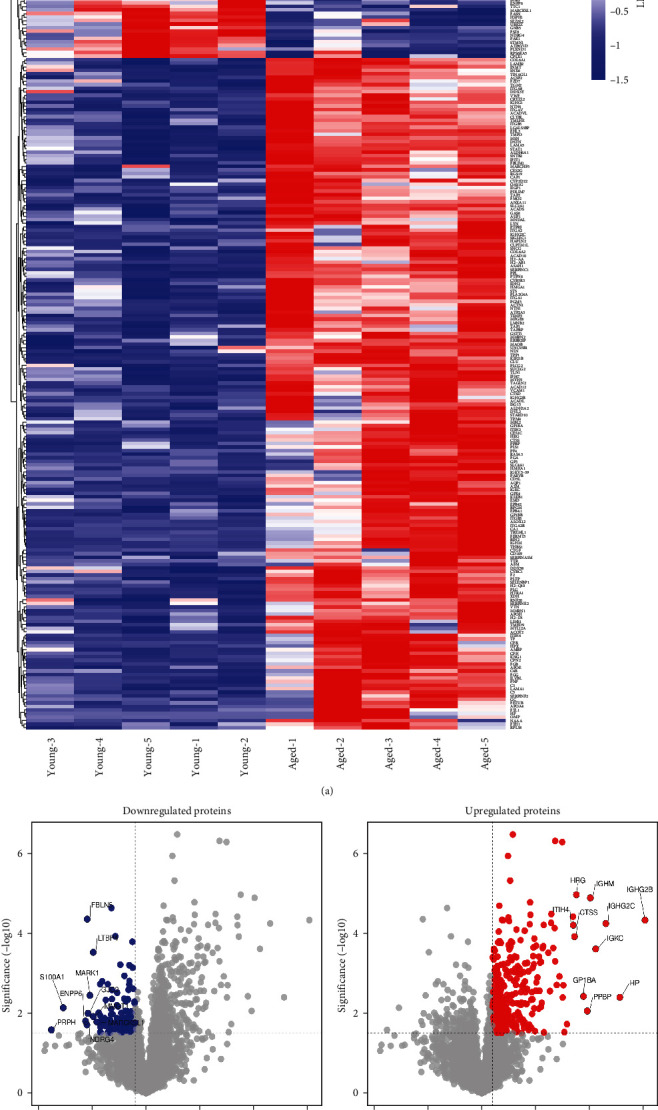
Significant differences between the proteomes of the CoW in young and aged mice. (a) Hierarchical clustering analysis (heatmap) generated using the unsupervised Euclidean distance of all differentially expressed proteins in all biological replicates. The *z*-scored LFQ intensity for individual mice are shown side by side. (b and c) Volcano plots for all proteins differentially expressed between groups, showing the –log10 statistical *p* value significance (*y*-axis) and the log2 fold change (*x*-axis). (b) Proteins whose levels are significantly decreased in the aged versus the young group (*p*  < 0.05) are shown in blue. (c) Proteins whose levels are significantly increased in the aged versus the young group (*p*  < 0.05) are shown in red. Top 10 up- and downregulated proteins (based on fold change) are labeled. The dotted line represents the cutoff, a measurement of protein expression fold change (log2) on the *X*-axis versus a measure of statistical significance (–log10 (*p* value)) on the *Y*-axis.

We generated a hierarchical cluster heatmap using the LFQ intensity of all significantly dysregulated proteins. This revealed a strong difference in the protein expression in the cerebral artery of the CoW between young and aged mice (Figure 2(a)). Among the 242 proteins, 189 were significantly upregulated and 53 downregulated (*Supplementary 4*). Volcano plots of all significantly down- or upregulated proteins are shown in Figures 2b) and 2(c). To understand the biological significance of the observed dysregulation, we analyzed the list of up- and downregulated proteins using Enrichr-KG bioinformatics webtool, with an FDR of *p*  < 0.05 for biological process. The processes driven by the 189 upregulated proteins included platelet degranulation (GO:0002576), regulated exocytosis (GO:0045055), negative regulation of blood coagulation (GO:0030195), and extracellular matrix organization (GO:0030198). Those driven by the 53 downregulated proteins contribute to intermediate filament bundle assembly (GO:0045110) and supramolecular fiber organization (GO:0097435).

Given that aging alters the mitochondrial proteome [46, 47], we mapped our data to the mouse MitoCarta mitochondria proteome database. Of 761 mitochondrial proteins identified, 548 were quantified. Among these, 21 proteins were significantly dysregulated in the aged CoW (Table 2), among 20 proteins were upregulated. All dysregulated mitochondrial proteins were involved in fatty acid oxidation (GO:0019395), fatty acid catabolism (GO:0009062), of fatty acid *β*-oxidation by acyl-CoA dehydrogenase (GO:0033539).

**Figure 3:**
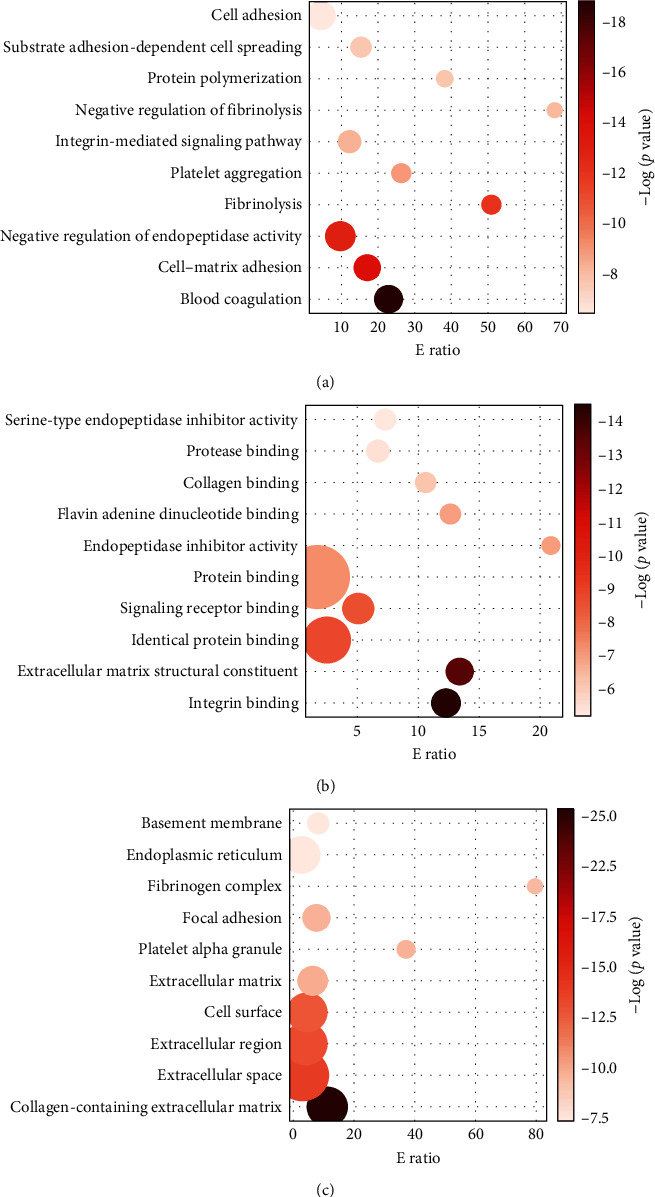
Gene ontology (GO) enrichment analysis of proteins that are dysregulated in the aged CoW. (a) GO biological process, (b) GO molecular function, and (c) GO cellular components. The top 10 significantly enriched GO terms are shown with their respective corrected *p* values and E ratios. Colored dots (dark to light) indicate enriched terms with their corrected *p* values, and size represents the E ratio for each enriched term. The analysis was performed using HemI 2.0 webtool.

A previous study had analyzed the proteomes of arteries of the CoW in 6-month-old mice [16]. It identified a total of 6,627 proteins and 2,188 with at least two unique high-scoring peptides. That study deployed two proteomics approaches, gel-free nano-LC-mass spectrometry (MS)/MS and gel-based GelLC-MS/MS with spectrometry. In our analysis, we used the Adaptive Focused Acoustics (AFA) technology to prepare the samples. Our analysis revealed an additional 3208 proteins not identified by previous study (*Supplementary 5*). By combining the previously identified proteins and those uniquely identified in our study, we created a CoW proteome database (*Supplementary 5)* for a list of 9835 CoW proteins. Gene enrichment analysis of the 3,208 novel proteins using the KEGG pathway revealed that the newly identified proteins are involved in metabolic pathways (*n* = 350), endocytosis (*n* = 75), MAPK signaling (*n* = 62), spliceosome (*n* = 56), and RNA transport (*n* = 54) (*Supplementary 6*). Furthermore, the current study detected additional proteins by KEGG pathways and GO analysis for cellular components in pathways previously implicated. (*Supplementary 6 and 7*). Further analyses were performed to identify proteins specific to vascular endothelial and smooth muscle cells. All 242 significantly dysregulated proteins were compared to our in-house generated human microvascular endothelial cell proteome (unpublished data) and published vascular smooth muscle proteome data [45]. We mapped 148 significantly dysregulated proteins to the human microvascular endothelial cell proteome, with 111 of these upregulated and the other 37 downregulated. Similarly, 22 significantly dysregulated proteins mapped to the vascular smooth muscle proteome, with 16 upregulated and 6 downregulated. This result suggests that aging-induced changes are more pronounced in endothelial than vascular smooth muscle cells (*Supplementary 8 and 9*). Gene ontology (GO) analysis of the dysregulated proteins revealed association (based on corrected *p*-value) for 33 GO terms for biological process, most prominently blood coagulation and cell–matrix adhesion and 22 GO terms for molecular function, such as integrin binding and extracellular matrix structural constituents (Figures 3(a) and 3(b)). Dysregulated proteins also enriched for 27 GO terms for cellular components, including collagen-containing extracellular matrix, extracellular space (Figure 3(c)).

### 3.2. Canonical Pathway and Upstream Regulator Analysis

**Figure 4:**
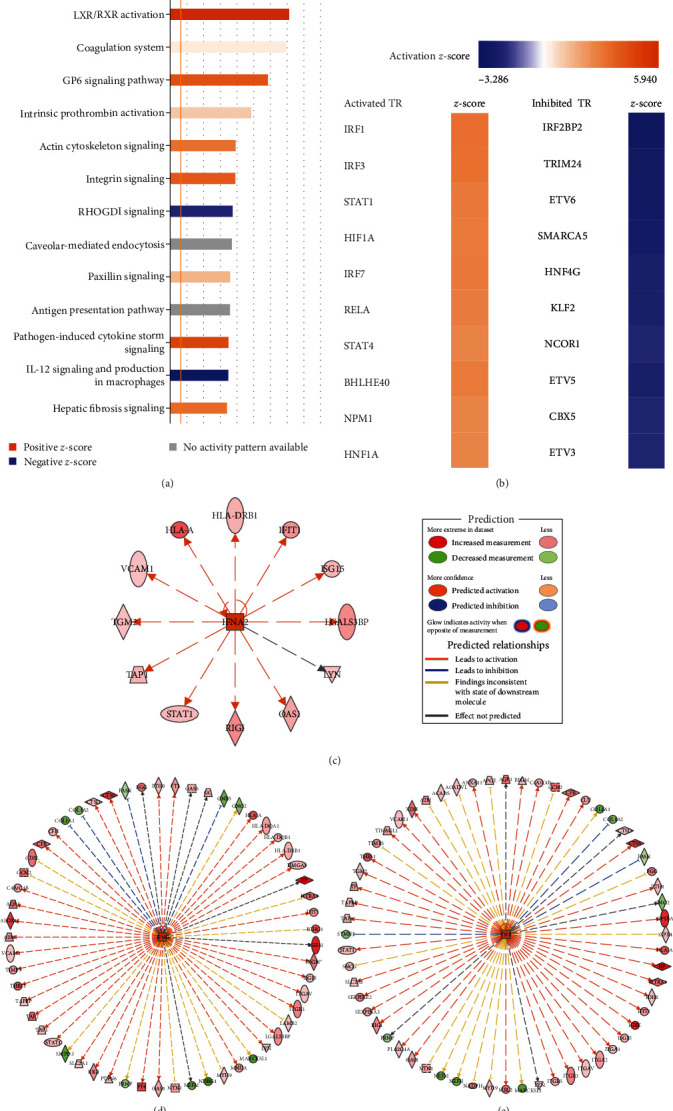
Ingenuity pathway analysis (IPA) of proteins that are dysregulated in the aged CoW. (a) Top 15 predicted canonical pathways based on *z*-score and B–H *p* value <0.05 (B–H *p* value was determined using Fischer's exact test and adjusted for multiple comparisons according to the method from Benjamini–Hochberg). A positive *z*-score implies activation (orange), and a negative *z*-score inhibition (blue), of the pathway; longer bars indicate stronger significance than the shorter bars (http://www.INGENUITY.com). (b) Heatmap of the top 10 transcription regulators predicted to be activated or inhibited based on *z*-score (>2 for activated and <–2 for inhibited). (c–e) Predicted activation or inhibition of inflammatory molecules in the aged CoW, and their gene networks, for (c) IFN*α*2, () IFN*γ*, and (e) TNF with their gene networks. Genes predicted, based on parameters described in (b), to be upregulated are marked in red, and those predicted to be downregulated are marked in green, as indicated in the prediction legend.

Canonical pathways that are altered in the aged CoW were identified by core analysis using the IPA software. Ninety-seven enriched canonical pathways were affected by applying the −log (*p* value) >2 threshold. The top 15 of the canonical pathways are shown in Figure 4(a) and *Supplementary 10*. Most of the activated canonical pathways contribute to inflammation, immune response, cytokine, and integrin-mediated signaling, and the top three were phagosome formation, neutrophil extracellular trap signaling, and integrin signaling. The inhibited pathway was IL-12 production with signaling in macrophages.

Next, we performed an analysis of upstream regulators including transcription regulators, ligand-dependent nuclear receptors, cytokines, and growth factors. Application of a *p* value of overlap <0.05 as a threshold revealed enrichment of 273 transcription regulators and 63 cytokines, including 29 that were activated and 10 that were inactivated. The top five activated transcription regulators were IRF1, IRF3, IRF7, STAT1, and BHLHE40. The top inhibited transcription regulators were IRF2BP2, TRIM24, ETV6, SMARCA5, and ETV5 (Figure 4(b)). Further reactome pathway analysis showed that most activated transcription regulators are involved in the JAK/STAT, PDGF, IFN*γ*, and interleukin signaling pathways (*Supplementary 11*). For the predicted cytokines, 19 were classified as activated and one as inhibited. The top three activated cytokines are IFN*γ*, TNF, and IFNA2, and the networks of differentially expressed genes regulated by these cytokines are shown in Figures 4(c), 4(d), and 4(e). The reactome pathway analysis revealed that the activated cytokines are involved in processing and signaling by IL1, IL4, IL6, IL10, and IL13 (*Supplementary 12*).

### 3.3. Impact Pathway Analysis

**Figure 5:**
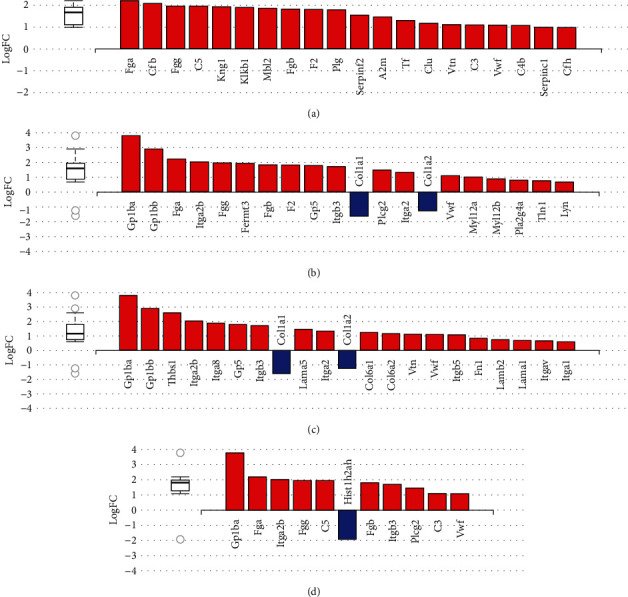
Impact pathway (iPathway) analysis of proteins that are dysregulated in the aged CoW. (a–d) Bar graphs of dysregulated genes that map to the top four pathways identified by iPathway analysis based on FDR-corrected *p* value significance: (a) complement and coagulation cascades, (b) platelet activation, (c) ECM–receptor interaction, and (d) neutrophil extracellular trap formation. All differentially expressed genes in each pathway were ranked based on their absolute value of log-fold change. Upregulated genes are shown in red, and downregulated genes in blue. The box-and-whisker plots on the left summarize the distributions of all the differentially expressed genes in their pathways. The box represents the 1^st^ quartile, median, and 3^rd^ quartile; circles represent the outliers (© Advaita Corporation 2023).

The differentially expressed proteins, fold-changes, and FDR-corrected *p* values were used as input for the iPathwayGuide webtool (https://ipathwayguide.advaitabio.com) [48, 49]. As opposed to other functional analysis tools, iPathwayGuide considers the position of the gene in each pathway and the type of interaction with other genes. The proteome in the aged CoW was enriched for 97 significantly affected pathways with an FDR-corrected *p* value threshold of <0.05 (*Supplementary 13*). Pathways with known or potential biological relevance to vascular disease included complement and coagulation cascades, platelet activation, ECM–receptor interaction, neutrophil extracellular trap formation (Figures 5(a), 5(b), 5(c), and 5(d)), and leukocyte transendothelial migration (*Supplementary 6*). Most of the dysregulated proteins were upregulated in those pathways. Also, of 27 dysregulated proteins involved in the metabolic process, most were upregulated in the aging CoW (*Supplementary 6*).

## 4. Discussion

In this study, we performed an unbiased, quantitative analysis of the proteomes in cerebral arteries of the CoW of young (2–3-month-old) and aged (18−20-month-old) mice to characterize the impact of aging on expression and relative abundance of proteins in cerebral arteries. The age ranges of the mice we analyzed (2–3 months and 18–20 months) correspond to approximately 16–20 and 56–60 years of age in humans, representing young adulthood and late middle age. Of note, the incidence of stroke and dementia is increasing in this age group at a faster rate in on older patients [50, 51]. Of 6,874 proteins that we identified in the CoW arteries, 242 proteins changed in abundance between these time points. Most of these proteins are known to contribute to blood coagulation, platelet dysfunction, altered extracellular matrix composition, and metabolic processes. Activated canonical pathways relate to inflammation and immune responses, including cytokine- and integrin-mediated signaling and activation of transcription factors (e.g., IRF1, IRF3, STAT1) and cytokines (e.g., IFN*α*, IFN*γ*, and TNF). Aging also affected the metabolism of CoW arteries, including increasing the abundance of proteins that catalyze various steps of fatty acid *β*-oxidation in the mitochondria. Our study adds to previous reports on the cerebral artery proteome, specifically in the CoW, by providing data on changes induced by aging.

A previous study by Badhwar and colleagues analyzed the CoW proteome in 6-month-old male mice of the same genotype [16]. Three thousand sixty-two of the proteins we detected overlapped with those reported in the previous study, and the five most abundant and significant in both datasets were Col6a1 (collagen alpha-1(VI) chain), Lama5 (laminin subunit alpha-5), Lamb2 (laminin subunit alpha-2), Myh9 (myosin-9), and Pdlim7 (PDZ and LIM domain protein 7). We detected 3,208 additional proteins that were not identified in the other study, many of them involved in metabolic pathways, endocytosis, and spliceosome. In our data set, 148 endothelial cell and 22 smooth muscle cell proteins were dysregulated during aging. Several highly dysregulated endothelial and smooth muscle cell proteins had previously been related to aging by methods other than proteomics. Among those that are endothelial cell lactadherin [52] and smooth muscle protein thrombospondin-1 [53]. Among those reduced were collagen *α*-1 and *α*-2 [54].

Changes in the lipid metabolism play an important role in the aging process [55, 56]. Our data demonstrate that various acyl-CoA dehydrogenases are upregulated and fatty acid synthase is downregulated with aging. A prior study reported that the relative abundance of glycolytic proteins was reduced in cerebral microvessels of 20-month-old versus 14-month-old mice [18]. Our study design does not allow us to attribute metabolic changes to specific cell types of the vascular wall. Of note, differential metabolic activity has been reported in cells of the vascular wall: differentiated smooth muscle cells in the intact vascular wall use oxidative phosphorylation to fuel vascular reactivity [57], whereas endothelial cells rely on glycolysis for their baseline metabolic needs and on mitochondrial respiration for specialized processes such as transendothelial migration [58]. How a shift toward fatty acid catalysis with aging as implicated by our data affects vascular wall physiology remains to be elucidated.

Whereas the proteomes of the cerebral macro- and microvasculature have been established, age-related changes are incompletely studied [16, 59, 60, 61]. One study combined samples from middle cerebral arteries and small mesenteric resistance arteries of 3- and 14-month-old C57Bl/6J mice, and the identified 31 proteins were significantly affected by age [23]. The most up- and downregulated proteins included myosin, Ig kappa chain V, laminin, and fibulin, of which other paralogs were also identified in our data set. Additional biological processes identified in our dataset included thrombosis, inflammation, and ECM remodeling all of which have been implicated in aging of the vasculature [1, 62, 63, 64].

Our study has several limitations. Firstly, we did not perform extensive validation of our data with an alternative method due to the limited quantity of protein in CoW samples. Secondly, our data were generated in only male mice, and therefore, the findings cannot be extrapolated to females without experimental confirmation. Lastly, we studied mice at only two ages, corresponding in humans to young adults and adults at about 60 years. An understanding of proteome changes associated with aging will require investigation of protein abundance at more advanced ages, corresponding to human age of 65–76 years and above. The current evidence is not sufficient to answer whether preventing stroke and cerebral hemorrhage in older versus younger patients will require additional or different vascular care. Further research on the progression of proteomic changes in the CoW of elderly patients is needed to produce the evidence base necessary to address this question.

## Figures and Tables

**Table 1:** List of all significantly dysregulated proteins in the aged cerebral arteries of the circle of Willis.

Gene symbol	Aged vs. young	Aged vs. young	Description
	Ratio	Adj *p* value	
**Ighg2b**	66.66551828	4.73215E-05	Ig gamma-2B chain C region
**Hp**	34.89256719	0.004009991	Haptoglobin
**Ighg2c**	24.35882266	5.75132E-05	Ig gamma-2A chain C region secreted form
**Igkc**	18.75882956	0.000248339	Ig kappa chain C region
**Ighm**	16.32061572	1.33228E-05	Ig mu chain C region
**Ppbp**	15.12449222	0.008796084	Chemokine subfamily B Cys-X-Cys
**Gp1ba**	13.72427612	0.003790987	Platelet glycoprotein Ib alpha chain
**Hrg**	11.40726077	1.09678E-05	Histidine-rich glycoprotein
**Ctss**	10.88691585	0.00012196	Cathepsin S
**Itih4**	10.52458675	6.32835E-05	Inter alpha-trypsin inhibitor, heavy chain 4
Alox12	10.46250419	3.87553E-05	Arachidonate 12-lipoxygenase, 12S-type
Igkv5-39	8.967052512	0.018812271	Immunoglobulin kappa variable 5-39
Omp	8.386840906	0.029903688	Olfactory marker protein
Inmt	8.174303124	0.002000071	Indolethylamine N-methyltransferase
Pf4	8.030857586	0.004054278	Platelet factor 4
Kif21b	7.932952781	5.30818E-07	Kinesin-like protein; kinesin-like protein KIF21B
Gp1bb	7.38145133	0.001208052	Platelet glycoprotein Ib beta chain
Pls1	6.889718542	0.001975398	Plastin-1
Bin2	6.613095243	0.000504555	Bridging integrator 2
Mfge8	6.612962788	4.97683E-07	Lactadherin
H2-Q10	6.526543968	7.46201E-05	H-2 class I histocompatibility antigen
Siglec1	6.301067047	0.000235007	Sialoadhesin
Thbs1	5.982939257	3.33739E-05	Thrombospondin-1
Ighg1	5.954976527	5.55551E-05	Ig gamma-1 chain C region secreted form
Ambp	5.796361108	0.002817534	Protein AMBP
Hapln2	5.548666339	0.000308422	Hyaluronan and proteoglycan link protein 2
Acad12	4.936774326	6.26472E-05	Acyl-Coenzyme A dehydrogenase family, member 12
Mmrn1	4.88161808	0.000211565	Multimerin-1
Ca1	4.792981547	0.000236503	Carbonic anhydrase 1
Tap2	4.692553495	0.005527926	Antigen peptide transporter 2
Ctgf	4.608094867	0.006950448	Connective tissue growth factor
Fga	4.607946828	0.000523753	Fibrinogen alpha chain
Htra1	4.486535794	0.000549359	Serine protease HTRA1
Parvb	4.431659336	0.002777844	Beta-parvin
Xdh	4.365774117	0.00038675	Xanthine dehydrogenase/oxidase
Itih2	4.349735907	0.007668215	Inter-alpha-trypsin inhibitor heavy chain H2
Cfb	4.208114199	0.001278187	Complement factor B
Cd5l	4.084971353	0.001702331	CD5 antigen-like
Itga2b	4.062124182	0.000206856	Integrin alpha-Iib
Ifi47	4.036832814	0.002744162	GTP-binding protein
Fgg	3.871935204	0.000136311	Fibrinogen gamma chain
C5	3.863614549	0.007582241	Complement C5
Treml1	3.822192815	0.000601194	Trem-like transcript 1 protein
Kng1	3.785356636	0.000227762	Kininogen-1
Fermt3	3.747891275	0.000375406	Fermitin family homolog 3
Aqp1	3.705874791	0.000736838	Aquaporin-1
Klkb1	3.697639792	0.004554924	Plasma kallikrein
Fetub	3.654414799	0.010465951	Fetuin-B
Itga8	3.642852133	0.002991624	Integrin alpha-8
Mbl2	3.599136489	0.023559542	Mannose-binding protein C
Stard10	3.560315331	0.004607595	PCTP-like protein
Fgb	3.497174362	3.49303E-05	Fibrinogen beta chain
F2	3.473122671	1.70411E-05	Prothrombin
Plg	3.444076352	5.98938E-05	Plasminogen
Gp5	3.409934696	0.019369444	Platelet glycoprotein V
Rnf20	3.392991109	0.022252636	E3 ubiquitin–protein ligase BRE1A
Serpine2	3.340653781	0.022945304	Glia-derived nexin
Iigp1	3.333595809	0.000905906	Interferon-inducible GTPase 1
Itgb3	3.239372079	0.000552809	Integrin beta-3
Tmlhe	3.222358649	0.002492668	Trimethyllysine dioxygenase, mitochondrial
Epb42	3.173321914	0.005881455	Erythrocyte membrane protein band 4.2
Clptm1l	3.143563952	0.005908087	Cleft lip and palate transmembrane protein 1-like protein
Ces2g	3.083184481	0.002974418	Carboxylic ester hydrolase
Ntn4	3.051043824	0.000566704	Netrin-4
Ttr	3.045211829	0.005730912	Transthyretin
Ptprj	3.03572178	0.001608062	Protein–tyrosine–phosphatase
Cpn2	3.02078478	8.72026E-05	Carboxypeptidase N subunit 2
Fzd7	3.008631706	0.016397719	Frizzled-7
Lgals3bp	2.928099387	0.000942326	Galectin-3-binding protein
Serpinf2	2.91585117	0.001917023	Alpha-2-antiplasmin
Ddx58	2.910652372	0.018381689	Probable ATP-dependent RNA helicase DDX58
Dvl2	2.872791632	0.014356497	Segment polarity protein dishevelled homolog DVL-2
Fmo2	2.832842297	0.002160965	Dimethylaniline monooxygenase [N-oxide-forming] 2
Tmed9	2.792226431	0.015067386	Transmembrane emp24 domain-containing protein 9
Hpx	2.782251325	0.005471984	Hemopexin
Plcg2	2.751975315	0.002958842	1-Phosphatidylinositol 4,5-bisphosphate phosphodiesterase gamma-2
Marchf5	2.732647206	0.006203988	E3 ubiquitin–protein ligase MARCH5
A2m	2.728685168	0.000149619	Alpha-2-macroglobulin
Lama5	2.717820077	0.001050255	Laminin subunit alpha-5
Ifit1	2.642962006	0.000754504	Interferon-induced protein with tetratricopeptide repeats 1
Mndal	2.642091048	0.011565165	Myeloid cell nuclear differentiation antigen-like protein
Afm	2.633061378	0.014467188	Afamin
Ftl1	2.599056601	0.00584123	Ferritin
Apoa4	2.581425347	0.01011995	Apolipoprotein A-IV
Naaa	2.538530455	0.009513648	N-Acylethanolamine-hydrolyzing acid amidase
Timp3	2.530152325	0.000673053	Metalloproteinase inhibitor 3
Itga2	2.480366751	0.005630863	Integrin alpha-2
Gstt1	2.470970426	0.000938159	Glutathione S-transferase theta-1
Ces1c	2.465262082	0.001387918	Carboxylesterase 1C
Tinagl1	2.447473015	0.000397469	Tubulointerstitial nephritis antigen-like
Tf	2.444700911	8.15272E-05	Serotransferrin
Ntn1	2.416211994	0.001976502	Netrin-1
Serpina3m	2.390641491	0.003191596	Serine protease inhibitor A3M
H2-Aa	2.371586782	0.000769635	H-2 class II histocompatibility antigen
Col6a1	2.336524996	0.000381413	Collagen alpha-1(VI) chain
Ppl	2.330225841	0.000943843	Periplakin
Cybc1	2.322454557	0.011354886	Cytochrome b-245 chaperone 1
Ddx3y	2.287527295	0.01633265	ATP-dependent RNA helicase DDX3Y
Apoh	2.277553783	0.001546615	Beta-2-glycoprotein 1
Clu	2.232244335	3.42981E-07	Clusterin
Oas1g	2.228205011	0.005674726	2-5-Oligoadenylate synthase 1A
Col6a2	2.203487662	0.003457091	Collagen alpha-2(VI) chain
Ilvbl	2.183603465	0.000171933	Acetolactate synthase-like protein
Slc4a1	2.181113505	0.013039114	Band 3 anion transport protein
Rasa3	2.160080575	0.000472971	Ras GTPase-activating protein 3
Pltp	2.142968315	0.000909461	Phospholipid transfer protein
Vtn	2.142822129	0.000453661	Vitronectin
Fhl3	2.124255306	0.000162154	Four and a half LIM domain protein 3
C3	2.123557247	0.000457569	Complement C3
Gas6	2.117577499	0.004967019	Growth arrest-specific protein 6
Vwf	2.111448907	0.00021769	von Willebrand factor
C4b	2.080584535	0.00358202	Complement C4-B
Itgb5	2.079615969	0.000406536	Integrin beta; integrin beta-5
Tpp1	2.077088054	4.86311E-06	Tripeptidyl-peptidase 1
H2-Ab1	2.069646406	0.001337074	H-2 class II histocompatibility antigen
H2-D1	2.040998513	0.000254324	H-2 class I histocompatibility antigen
Lmnb2	2.028829222	5.00737E-05	Lamin-B2
Cryzl2	1.991607158	0.000199507	Quinone oxidoreductase-like protein 2
Myl12a	1.9851367	0.008506506	Myosin, light chain 12A, regulatory, nonsarcomeric
Asah1	1.984775602	4.07193E-05	Acid ceramidase
Gc	1.978554901	0.010282938	Vitamin D-binding protein
Serpinc1	1.966131897	1.1789E-06	Antithrombin-III
Cfh	1.962196235	0.000555113	Complement factor H
Hmha1	1.961464817	0.01197487	Minor histocompatibility protein HA-1
Ptpn6	1.945732031	0.000912445	Tyrosine–protein phosphatase nonreceptor type 6
Tap1	1.927080522	0.005646196	Antigen peptide transporter 1
Pgm5	1.911888411	0.003544745	Phosphoglucomutase-like protein 5
Rgs19	1.88166638	0.001125179	Regulator of G-protein signaling 19
Isg15	1.871893415	0.007719428	Ubiquitin-like protein ISG15
Hmga1	1.865502588	0.007225555	High mobility group protein HMG-I/HMG-Y
Cd109	1.85605882	0.004469744	CD109 antigen
Stat1	1.841594596	0.000806543	Signal transducer and activator of transcription
Fth1	1.787782221	0.007409893	Ferritin heavy chain
Sncg	1.777112844	0.006810918	Gamma-synuclein
Epb4.1	1.775555826	0.002137504	Protein 4.1
Tapbp	1.768951714	0.002015833	Tapasin
Acad10	1.757974921	0.000597312	Acyl-CoA dehydrogenase family member 10
Slc2a1	1.738492769	4.78066E-05	Solute carrier family 2, facilitated glucose transporter member 1
Unc93b1	1.738447146	0.006076412	Protein unc-93 homolog B1
Pla2g4a	1.711897398	0.004417992	Cytosolic phospholipase A2; phospholipase A2; lysophospholipase
Rpl38	1.689304819	0.002103458	60S ribosomal protein L38
Snx6	1.688542067	0.006459252	Sorting nexin-6; sorting nexin-6, N-terminally processed
Tagln2	1.684630124	4.66547E-05	Transgelin-2
Tgm2	1.67393931	0.007338137	Protein-glutamine gamma-glutamyltransferase 2
Tln1	1.672849507	0.001638181	Talin-1
Mmrn2	1.660038463	0.004924877	Multimerin-2
Lamb2	1.656700349	0.000614091	Laminin subunit beta-2
Sts	1.649457642	0.002580989	Steryl-sulfatase
Apoe	1.64151551	0.000176626	Apolipoprotein E
Nln	1.625233461	0.003303669	Neurolysin, mitochondrial
Lims1	1.619314773	0.001486289	LIM and senescent cell antigen-like-containing domain protein 1
Atp2a3	1.612991085	0.004937198	Calcium-transporting ATPase
Bpgm	1.607125054	0.003459709	Bisphosphoglycerate mutase
Emd	1.592862045	0.004855339	Emerin
Lama1	1.588896982	0.000162466	Laminin subunit alpha-1
Pnp	1.585908068	0.000158637	Purine nucleoside phosphorylase
Pdlim7	1.584881024	0.001721542	PDZ and LIM domain protein 7
Lyn	1.58293524	0.002998901	Tyrosine–protein kinase Lyn
Tpm4	1.57964759	0.003864778	Tropomyosin alpha-4 chain
Clybl	1.578625853	0.00181524	Citrate lyase subunit beta-like protein, mitochondrial
Itgav	1.573671868	2.07186E-05	Integrin alpha-V
Aup1	1.570431278	0.00455977	Ancient ubiquitous protein 1
Acsf2	1.55229592	9.12428E-05	Acyl-CoA synthetase family member 2, mitochondrial
Acot2	1.548522324	0.002274296	Acyl-coenzyme A thioesterase 2, mitochondrial
Fblim1	1.541751322	0.001064217	Filamin-binding LIM protein 1
Cyp2d22	1.531931179	0.001399651	Cytochrome P450
Acads	1.525239982	0.00017012	Short-chain specific acyl-CoA dehydrogenase, mitochondrial
Erbb2ip	1.524461013	0.00176213	Protein LAP2
Actn1	1.51372641	0.002847927	Actinin, alpha 1
Sntb2	1.512894916	0.001345227	Beta-2-syntrophin
Maob	1.50253334	0.000387397	Amine oxidase [flavin-containing] B
Myh9	1.501865243	0.000657315	Myosin-9
Aldh3a2	1.496711213	0.003306956	Aldehyde dehydrogenase
Itga1	1.494774467	0.002632206	Integrin alpha-1
Suclg2	1.492771547	0.001575535	Succinyl-CoA ligase [GDP-forming] subunit beta, mitochondrial
Gpx4	1.492403722	0.000743093	Phospholipid hydroperoxide glutathione peroxidase
Aldh6a1	1.490948703	0.000749543	Methylmalonate-semialdehyde dehydrogenase [acylating], mitochondrial
Ctsd	1.480479438	0.000894481	Cathepsin D
Selenbp1	1.4766641	0.001474392	Selenium-binding protein 1
Acadl	1.453578277	0.001443625	Long-chain specific acyl-CoA dehydrogenase, mitochondrial
Msn	1.449076483	0.001124149	Moesin
Cyb5r3	1.448873041	0.001061495	NADH-cytochrome b5 reductase 3
Idh2	1.428476734	0.000729345	Isocitrate dehydrogenase [NADP], mitochondrial
Dstn	1.402667727	0.000273143	Destrin
Cap1	1.396822169	0.000863081	Adenylyl cyclase-associated protein 1
Acadvl	1.387752674	0.000255319	Very long-chain specific acyl-CoA dehydrogenase, mitochondrial
Anxa11	1.370822966	0.000248575	Annexin A11
Vcam1	1.370751924	0.000273871	Vascular cell adhesion protein 1
Tmpo	1.361661586	0.000454738	Lamina-associated polypeptide 2, isoforms beta/delta/epsilon/gamma
Erp29	0.710365102	0.000730458	Endoplasmic reticulum resident protein 29
Klc1	0.703682714	0.000164282	Kinesin light chain 1
Cltb	0.651639722	0.001579567	Clathrin light chain B
Ube2m	0.650270147	0.000633063	NEDD8-conjugating enzyme Ubc12
Rpl27a	0.648322571	0.001930202	60S ribosomal protein L27a
Fasn	0.637251331	0.00260969	Fatty acid synthase
Hsph1	0.629128665	0.004587015	Heat shock protein 105 kDa
Rab5a	0.620922667	0.004438894	Ras-related protein Rab-5A
Sh3glb2	0.595426397	0.005022801	Endophilin-B2
Wdr37	0.573666034	0.001164147	WD repeat-containing protein 37
Prnp	0.570914099	0.004361925	Major prion protein
Pak1	0.529453537	0.009880754	Nonspecific serine/threonine protein kinase
Praf2	0.516657501	0.000616254	PRA1 family protein 2
Gng2	0.487241928	0.006759911	Guanine nucleotide-binding protein G(I)/G(S)/G(O) subunit gamma-2
Stmn1	0.477988027	0.013523114	Stathmin
P3h1	0.476813142	0.003088627	Prolyl 3-hydroxylase 1
Cadm4	0.474348484	0.006374039	Cell adhesion molecule 4
Atp6v1d	0.473250535	0.013416516	V-type proton ATPase subunit D
Nrip2	0.45442483	0.000119907	Nuclear receptor-interacting protein 2
Rps6ka5	0.425688	0.010645407	Ribosomal protein S6 kinase alpha-5
Brsk2	0.417014066	0.004420589	Serine/threonine–protein kinase BRSK2
Col1a2	0.416266592	0.002618919	Collagen alpha-2(I) chain
Mag	0.412234897	0.017391594	Myelin-associated glycoprotein
Hist1h1b	0.411385022	2.3559E-05	Histone H1.5
Tsr2	0.408642803	0.007929398	Pre-rRNA-processing protein TSR2 homolog
Cnot3	0.408236584	0.010875511	CCR4-NOT transcription complex subunit 3
Sez6l2	0.406706428	0.018228379	Seizure 6-like protein 2
Ttc1	0.398489831	0.014869363	Tetratricopeptide repeat protein 1
Ube2z	0.396026315	0.015907645	Ubiquitin-conjugating enzyme E2 Z
Gnb5	0.38585012	0.014488818	Guanine nucleotide-binding protein subunit beta-5
Fip1l1	0.374304663	0.001892411	Pre-mRNA 3-end-processing factor FIP1
Bcas1	0.355402569	0.004685618	Breast carcinoma-amplified sequence 1 homolog
Thsd4	0.344288495	0.013389635	Thrombospondin type-1 domain-containing protein 4
Nefh	0.338924312	0.01595098	Neurofilament heavy polypeptide
Nefm	0.335538951	0.01508474	Neurofilament medium polypeptide
Cplx1	0.328591046	0.01580599	Complexin-1
Col1a1	0.324495138	0.001599379	Collagen alpha-1(I) chain
Plxnd1	0.31886202	0.013188994	Plexin-D1
Klc2	0.312373752	0.017118233	Kinesin light chain 2
Smpd3	0.310345454	0.022347603	Sphingomyelin phosphodiesterase 3
Nefl	0.307053308	0.011373004	Neurofilament light polypeptide
Aamdc	0.306891418	0.001880335	Mth938 domain-containing protein
Fsd1	0.295618383	0.009786318	Fibronectin type III and SPRY domain-containing protein 1
**Marcksl1**	0.28259879	0.016563876	MARCKS-related protein
**Mmgt1**	0.257837339	0.012186557	Membrane magnesium transporter 1
**Ltbp4**	0.257414254	0.000300755	Latent-transforming growth factor beta-binding protein 4
**Mark1**	0.236298072	0.003601724	Serine/threonine–protein kinase MARK1
**Gjc3**	0.224793288	0.010090383	Gap junction gamma-3 protein
**Fbln5**	0.220280561	4.49075E-05	Fibulin-5
**Ndrg4**	0.218862031	0.019650245	Protein NDRG4
**Enpp6**	0.211374284	0.015808187	Ectonucleotide pyrophosphatase/phosphodiesterase family member 6
**S100a1**	0.119034494	0.007336224	Protein S100-A1
**Prph**	0.087889623	0.02609444	Peripherin

Top 10 upregulated and downregulated proteins are in bold.

**Table 2:** List of all significantly dysregulated mitochondrial proteins in the aged cerebral arteries of the circle of Willis.

Gene names	Aged vs. young	Aged vs. young	Description
	Ratio	Adj *p* value	
Acad10	1.757974921	0.000597312	Acyl-CoA dehydrogenase family member 10
Acad12	4.936774326	6.26472E-05	Acyl-coenzyme A dehydrogenase family, member 12
Acadl	1.453578277	0.001443625	Long-chain specific acyl-CoA dehydrogenase, mitochondrial
Acads	1.525239982	0.00017012	Short-chain specific acyl-CoA dehydrogenase, mitochondrial
Acadvl	1.387752674	0.000255319	Very long-chain specific acyl-CoA dehydrogenase, mitochondrial
Acot2	1.548522324	0.002274296	Acyl-coenzyme A thioesterase 2, mitochondrial
Acsf2	1.55229592	9.12428E-05	Acyl-CoA synthetase family member 2, mitochondrial
Aldh3a2	1.496711213	0.003306956	Aldehyde dehydrogenase; fatty aldehyde dehydrogenase
Aldh6a1	1.490948703	0.000749543	Methylmalonate-semialdehyde dehydrogenase [acylating], mitochondrial
Clybl	1.578625853	0.00181524	Citrate lyase subunit beta-like protein, mitochondrial
Cryzl2	1.991607158	0.000199507	Quinone oxidoreductase-like protein 2
Cyb5r3	1.448873041	0.001061495	NADH-cytochrome b5 reductase 3
Fasn	0.637251331	0.00260969	Fatty acid synthase
Fth1	1.787782221	0.007409893	Ferritin heavy chain; ferritin heavy chain, N-terminally processed
Gpx4	1.492403722	0.000743093	Phospholipid hydroperoxide glutathione peroxidase, mitochondrial
Idh2	1.428476734	0.000729345	Isocitrate dehydrogenase [NADP], mitochondrial
Maob	1.50253334	0.000387397	Amine oxidase [flavin-containing] B
Marchf5	2.732647206	0.006203988	E3 ubiquitin-protein ligase MARCH5
Nln	1.625233461	0.003303669	Neurolysin, mitochondrial
Suclg2	1.492771547	0.001575535	Succinyl-CoA ligase [GDP-forming] subunit beta, mitochondrial
Tmlhe	3.222358649	0.002492668	Trimethyllysine dioxygenase, mitochondrial

## Data Availability

The mass spectrometry proteomics data have been deposited in the ProteomeXchange Consortium via the PRIDE partner repository [29] with the dataset identifier PXD043001 (website, http://www.ebi.ac.uk/pride; username, reviewer_pxd043001@ebi.ac.uk; and password, VkbRim9h).

## Supplementary Materials

Supplementary 1 The list of all Identified proteins from young and aged cerebral arteries of the circle of Willis.

Supplementary 2 The list of all quantified proteins from young and aged cerebral arteries of the circle of Willis.

Supplementary 3 The proteins quantified at least with 2 unique peptides in bold.

Supplementary 4 List of all significantly dysregulated proteins in the aged cerebral arteries of the circle of Willis.

Supplementary 5 List of all identified proteins from the circle of Willis by Badhwar et al. and of proteins uniquely in the current study after removing Rik proteins and proteins identified without gene and protein names.

Supplementary 6 Figure 1: KEGG pathway analysis of proteins identified only in this study (*n* = 3208). Figure 2: gene ontology and KEGG pathway analysis of all proteins identified in Badhwar et al. and in this study. Figure 3: impact pathway analysis of proteins differentially expressed in the aged versus young CoW.

Supplementary 7 Top KEGG pathways and GO cellular components enriched in two datasets based on all identified proteins using ExpressAnalyst webtool.

Supplementary 8 The list of identified endothelial cell proteins.

Supplementary 9 The list of identified vascular smooth muscle cell proteins.

Supplementary 10 The list of all activated and inhibited canonical pathways with overlapped molecules in the aged circle of Willis.

Supplementary 11 The list of all activated and inhibited transcription regulators with target molecules in the aged circle of Willis.

Supplementary 12 The list of all activated and inhibited cytokines with target molecules in the aged circle of Willis.

Supplementary 13 The list of all impacted pathways identified using iPathwayGuide tool.

## References

[1] Ungvari Z., Kaley G., de Cabo R., Sonntag W. E., Csiszar A. (2010). Mechanisms of vascular aging: new perspectives. *The Journals of Gerontology Series A: Biological Sciences and Medical Sciences*.

[2] Ungvari Z., Toth P., Tarantini S. (2021). Hypertension-induced cognitive impairment: from pathophysiology to public health. *Nature Reviews Nephrology*.

[3] Vasilevko V., Passos G. F., Quiring D. (2010). Aging and cerebrovascular dysfunction: contribution of hypertension, cerebral amyloid angiopathy, and immunotherapy. *Annals of the New York Academy of Sciences*.

[4] Diaz-Otero J. M., Garver H., Fink G. D., Jackson W. F., Dorrance A. M. (2016). Aging is associated with changes to the biomechanical properties of the posterior cerebral artery and parenchymal arterioles. *American Journal of Physiology-Heart and Circulatory Physiology*.

[5] Peisker T., Bartoš A., Skoda O., Ibrahim I., Kalvach P. (2010). Impact of aging on cerebral vasoregulation and parenchymal integrity. *Journal of The Neurological Sciences*.

[6] Uston C. (2005). NEUROwords Dr. Thomas Willis’ famous eponym: the circle of Willis. *Journal of the History of the Neurosciences*.

[7] Alpers B. J., Berry R. G., Paddison R. M. (1959). Anatomical studies of the circle of Willis in normal brain. *Archives of Neurology And Psychiatry*.

[8] Hamel E. (2006). Perivascular nerves and the regulation of cerebrovascular tone. *Journal of Applied Physiology*.

[9] Karatas A., Coban G., Cinar C., Oran I., Uz A. (2015). Assessment of the circle of willis with cranial tomography angiography. *Medical Science Monitor*.

[10] Kalaria R. N. (1996). Cerebral vessels in ageing and Alzheimer’s disease. *Pharmacology & Therapeutics*.

[11] Mandalà M., Cipolla M. J. (2021). Aging-related structural and functional changes in cerebral arteries: caloric restriction (CR) intervention. *Journal of Vascular Medicine & Surgery*.

[12] Nixon A. M., Gunel M., Sumpio B. E. (2010). The critical role of hemodynamics in the development of cerebral vascular disease. *Journal of Neurosurgery*.

[13] Wijesinghe P., Steinbusch H. W. M., Shankar S. K., Yasha T. C., De Silva K. R. D. (2020). Circle of Willis abnormalities and their clinical importance in ageing brains:a cadaveric anatomical and pathological study. *Journal of Chemical Neuroanatomy*.

[14] Stoquart-ElSankari S., Balédent O., Gondry-Jouet C., Makki M., Godefroy O., Meyer M.-E. (2007). Aging effects on cerebral blood and cerebrospinal fluid flows. *Journal of Cerebral Blood Flow & Metabolism*.

[15] Hoksbergen A. W. J., Legemate D. A., Csiba L., Csáti G., Síró P., Fülesdi B. (2003). Absent collateral function of the circle of Willis as risk factor for ischemic stroke. *Cerebrovascular Diseases*.

[16] Badhwar A. P., Stanimirovic D. B., Hamel E., Haqqani A. S. (2014). The proteome of mouse cerebral arteries. *Journal of Cerebral Blood Flow & Metabolism*.

[17] Nyúl-Tóth Á., Tarantini S., DelFavero J. (2021). Demonstration of age-related blood-brain barrier disruption and cerebromicrovascular rarefaction in mice by longitudinal intravital two-photon microscopy and optical coherence tomography. *American Journal of Physiology-Heart and Circulatory Physiology*.

[18] Chandra P. K., Cikic S., Rutkai I. (2022). Effects of aging on protein expression in mice brain microvessels: ROS scavengers, mRNA/protein stability, glycolytic enzymes, mitochondrial complexes, and basement membrane components. *GeroScience*.

[19] Lowerison M. R., Sekaran N. V. C., Zhang W. (2022). Aging-related cerebral microvascular changes visualized using ultrasound localization microscopy in the living mouse. *Scientific Reports*.

[20] Sakamuri S. S. V. P., Sure V. N., Kolli L. (2022). Aging related impairment of brain microvascular bioenergetics involves oxidative phosphorylation and glycolytic pathways. *Journal of Cerebral Blood Flow & Metabolism*.

[21] Nordon I., Brar R., Hinchliffe R., Cockerill G., Loftus I., Thompson M. (2009). The role of proteomic research in vascular disease. *Journal of Vascular Surgery*.

[22] Alaaeddine R., Fayad M., Nehme E., Bahmad H. F., Kobeissy F. (2017). The Emerging role of proteomics in precision medicine: applications in neurodegenerative diseases and neurotrauma. *Advances in Experimental Medicine and Biology*.

[23] Rabaglino M. B., Wakabayashi M., Pearson J. T., Jensen L. J. (2021). Effect of age on the vascular proteome in middle cerebral arteries and mesenteric resistance arteries in mice. *Mechanisms of Ageing and Development*.

[24] Hur J. C., Blaise R., Limon I. (2016). Protocol for isolating the mouse circle of Willis. *Journal of Visualized Experiments*.

[25] Giansanti P., Samaras P., Bian Y. (2022). Mass spectrometry-based draft of the mouse proteome. *Nature Methods*.

[26] Hughes C. S., Foehr S., Garfield D. A., Furlong E. E., Steinmetz L. M., Krijgsveld J. (2014). Ultrasensitive proteome analysis using paramagnetic bead technology. *Molecular Systems Biology*.

[27] Cox J., Mann M. (2008). MaxQuant enables high peptide identification rates, individualized p.p.b.-range mass accuracies and proteome-wide protein quantification. *Nature Biotechnology*.

[28] Cox J., Hein M. Y., Luber C. A., Paron I., Nagaraj N., Mann M. (2014). Accurate proteome-wide label-free quantification by delayed normalization and maximal peptide ratio extraction, termed MaxLFQ. *Molecular & Cellular Proteomics*.

[29] Vizcaíno J. A., Côté R. G., Csordas A. (2013). The PRoteomics IDEntifications (PRIDE) database and associated tools: status in 2013. *Nucleic Acids Research*.

[30] Azimzadeh O., Subramanian V., Sievert W. (2021). Activation of PPAR*α* by fenofibrate attenuates the effect of local heart high dose irradiation on the mouse cardiac proteome. *Biomedicines*.

[31] Philipp J., Sievert W., Azimzadeh O. (2020). Data independent acquisition mass spectrometry of irradiated mouse lung endothelial cells reveals a STAT-associated inflammatory response. *International Journal of Radiation Biology*.

[32] Subramanian V., Borchard S., Azimzadeh O. (2018). PPAR*α* is necessary for radiation-induced activation of noncanonical TGF*β* signaling in the heart. *Journal of Proteome Research*.

[33] Zhao T., Wang Z. (2022). GraphBio: a shiny web app to easily perform popular visualization analysis for omics data. *Frontiers in Genetics*.

[34] Guan D., Tian H. (2017). Integrated network analysis to explore the key genes regulated by parathyroid hormone receptor 1 in osteosarcoma. *World Journal of Surgical Oncology*.

[35] Goedhart J., Luijsterburg M. S. (2020). VolcaNoseR is a web app for creating, exploring, labeling and sharing volcano plots. *Scientific Reports*.

[36] Ning W., Wei Y., Gao L. (2022). HemI 2.0: an online service for heatmap illustration. *Nucleic Acids Research*.

[37] Xie Z., Bailey A., Kuleshov M. V. (2021). Gene set knowledge discovery with Enrichr. *Current Protocols*.

[38] Krämer A., Green J., Pollard J., Tugendreich S. (2014). Causal analysis approaches in ingenuity pathway analysis. *Bioinformatics*.

[39] Yip L., Alkhataybeh R., Taylor C., Fuhlbrigge R., Fathman C. G. (2022). Identification of novel disease-relevant genes and pathways in the pathogenesis of type 1 diabetes: a potential defect in pancreatic iron homeostasis. *Diabetes*.

[40] Faranda A. P., Shihan M. H., Wang Y., Duncan M. K. (2021). The aging mouse lens transcriptome. *Experimental Eye Research*.

[41] Ashburner M., Ball C. A., Blake J. A. (2000). Gene ontology: tool for the unification of biology. *Nature Genetics*.

[42] Szklarczyk D., Gable A. L., Nastou K. C. (2021). The STRING database in 2021: customizable protein-protein networks, and functional characterization of user-uploaded gene/measurement sets. *Nucleic Acids Research*.

[43] von Mering C., Huynen M., Jaeggi D., Schmidt S., Bork P., Snel B. (2003). STRING: a database of predicted functional associations between proteins. *Nucleic Acids Research*.

[44] Rath S., Sharma R., Gupta R. (2021). MitoCarta3.0: an updated mitochondrial proteome now with sub-organelle localization and pathway annotations. *Nucleic Acids Research*.

[45] Rocchiccioli S., Citti L., Boccardi C. (2010). A gel-free approach in vascular smooth muscle cell proteome: perspectives for a better insight into activation. *Proteome Science*.

[46] Ubaida-Mohien C., Lyashkov A., Gonzalez-Freire M. (2019). Discovery proteomics in aging human skeletal muscle finds change in spliceosome, immunity, proteostasis and mitochondria. *Elife*.

[47] Ingram T., Chakrabarti L. (2016). Proteomic profiling of mitochondria: what does it tell us about the ageing brain?. *Aging (Albany NY)*.

[48] Draghici S., Khatri P., Tarca A. L. (2007). A systems biology approach for pathway level analysis. *Genome Research*.

[49] Tarca A. L., Draghici S., Khatri P. (2009). A novel signaling pathway impact analysis. *Bioinformatics*.

[50] Alzheimers Association (2022). Alzheimer’s disease facts and figures. *Alzheimer’s & Dementia*.

[51] Bako A. T., Pan A., Potter T. (2022). Contemporary trends in the nationwide incidence of primary intracerebral hemorrhage. *Stroke*.

[52] Peng S., Glennert J., Westermark P. (2005). Medin-amyloid: a recently characterized age-associated arterial amyloid form affects mainly arteries in the upper part of the body. *Amyloid-International Journal of Experimental and Clinical Investigation*.

[53] Isenberg J. S., Roberts D. D. (2020). Thrombospondin-1 in maladaptive aging responses: a concept whose time has come. *American Journal of Physiology-Cell Physiology*.

[54] Hwang S. J., Ha G.-H., Seo W.-Y., Kim C. K., Kim K. J., Lee S. B. (2020). Human collagen alpha-2 type I stimulates collagen synthesis, wound healing, and elastin production in normal human dermal fibroblasts (HDFs). *BMB Reports*.

[55] Johnson A. A., Stolzing A. (2019). The role of lipid metabolism in aging, lifespan regulation, and age-related disease. *Aging Cell*.

[56] Mutlu A. S., Duffy J., Wang M. C. (2021). Lipid metabolism and lipid signals in aging and longevity. *Developmental Cell*.

[57] Barron J. T., Kopp S. J., Tow J., Parrillo J. E. (1994). Fatty acid, tricarboxylic acid cycle metabolites, and energy metabolism in vascular smooth muscle. *The American Journal of Physiology*.

[58] Ibrahim A., Yucel N., Kim B., Arany Z. (2020). Local mitochondrial ATP production regulates endothelial fatty acid uptake and transport. *Cell Metabolism*.

[59] Bisen S., Kakhniashvili D., Johnson D. L., Bukiya A. N. (2019). Proteomic analysis of baboon cerebral artery reveals potential pathways of damage by prenatal alcohol exposure. *Molecular & Cellular Proteomics*.

[60] Cuadrado E., Rosell A., Colomé N. (2010). The proteome of human brain after ischemic stroke. *Journal of Neuropathology & Experimental Neurology*.

[61] Müller A. H., Edwards A. V. G., Larsen M. R. (2017). Proteomic expression changes in large cerebral arteries after experimental subarachnoid hemorrhage in rat are regulated by the MEK-ERK1/2 pathway. *Journal of Molecular Neuroscience*.

[62] Mammoto A., Matus K., Mammoto T. (2022). Extracellular matrix in aging aorta. *Frontiers in Cell and Developmental Biology*.

[63] Trott D. W., Fadel P. J. (2019). Inflammation as a mediator of arterial ageing. *Experimental Physiology*.

[64] Mammen E. F., Suzuki S., Haas S. (2002). Aging and thrombosis. *Seminars in Thrombosis and Hemostasis*.

