# Supplementary material for: Changes in the Proteome of the Circle of Willis during Aging Reveal Signatures of Vascular Disease
Source: Oxid Med Cell Longev. 2024 Jun 26;2024:4887877. doi: 10.1155/2024/4887877 (PMC11221951; doi:10.1155/2024/4887877)
Supplement: Supplementary 6 — Figure 1: KEGG pathway analysis of proteins identified only in this study (n = 3208). Figure 2: gene ontology and KEGG pathway analysis of all proteins identified in Badhwar et al. and in this study. Figure 3: impact pathway analysis of proteins differentially expressed in the aged versus young CoW. [file 4887877.f6.docx]

**Changes in the Proteome of the Circle of Willis During Aging Reveal Signatures of Vascular Disease**

**Vikram Subramanian ^1^, Denise Juhr ^1^, Lydia S. Johnson ^1^, Justin B. Yem ^1^, Piero Giansanti ^2^, Isabella M. Grumbach MD, PhD ^1,3,4*^**

^1^ Abboud Cardiovascular Research Center, Department of Internal Medicine, Carver College of Medicine, University of Iowa

^2^ Bavarian Center for Biomolecular Mass Spectrometry (BayBioMS@MRI), Technical University of Munich, Munich, Germany

^3^ Free Radical and Radiation Biology Program, Department of Radiation Oncology, Carver College of Medicine, University of Iowa

^4^ Iowa City VA Healthcare System, Iowa City, IA


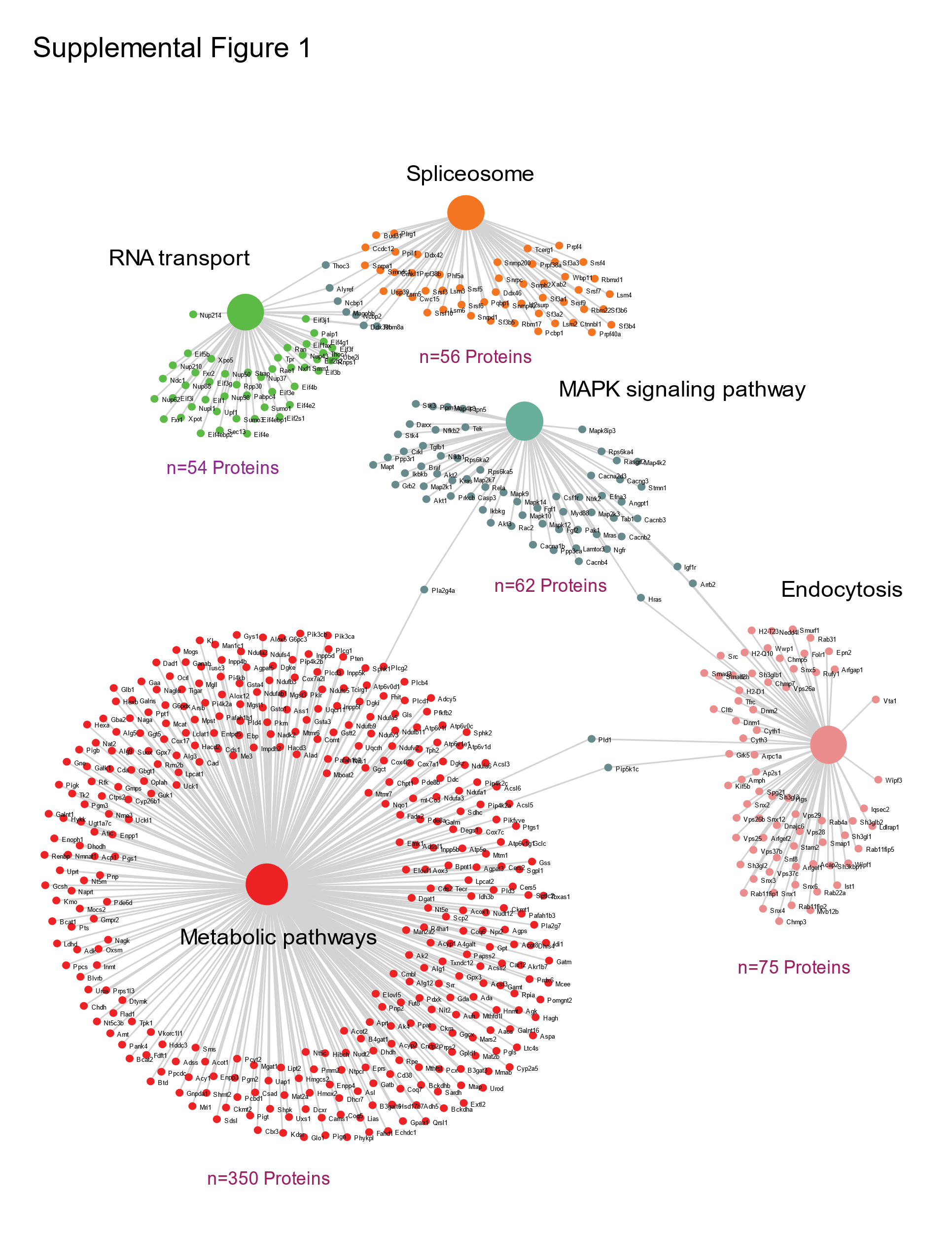


**Figure S1: KEGG pathway analysis of proteins newly identified in this study (n=3208)**. The top five enriched KEGG pathways (metabolic, endocytosis, MAPK signaling, spliceosome, and RNA transport) with their associated proteins. The enriched KEGG pathway terms are represented as center nodes and the proteins associated with the pathways as nearby small circles. Green circles represent the proteins shared between KEGG pathways.


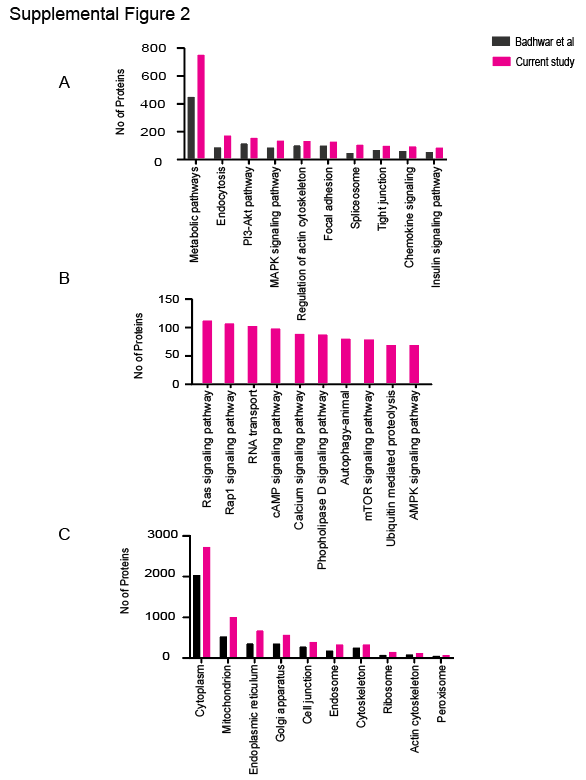


**Figure S2: Gene ontology and KEGG pathway analysis of all proteins identified in Badhwar *et al*. and this study.** (A) Number of proteins in the 10 most abundantly represented KEGG pathways in both datasets. (B) Number of proteins in the selected KEGG pathways enriched in the current study only. (C) Number of proteins in the 10 most abundantly represented cellular components by Gene ontology analysis in both datasets. Black bars represent pathways that were enriched only in Badhwar *et al*. and magenta bars represent pathways that were enriched only in the current study.


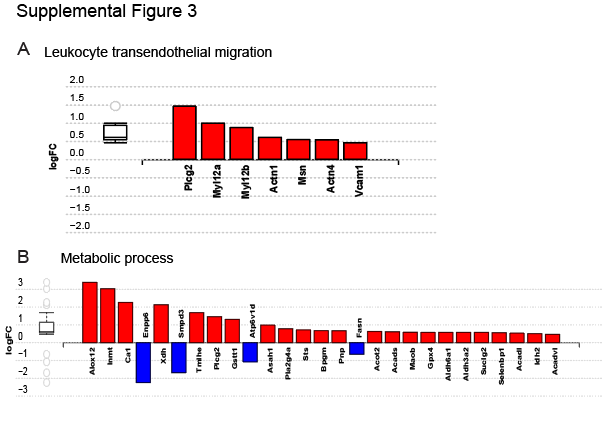


**Figure S3: Impact pathway analysis of proteins differentially expressed in the aged versus young CoW.** (A, B) Bar graphs of differentially expressed genes that map to two pathways identified by impact pathway analysis based on FDR-corrected p-value significance: (A) leukocyte transendothelial migration, and (B) metabolic processes. For each pathway, all the differentially expressed genes are ranked based on their absolute value of log-fold change. Upregulated genes are shown in red and downregulated genes in blue. The box-and-whisker plots on the left summarize the distributions of all differentially expressed genes in each pathway. The box represents the 1^st^ quartile, the median, and the 3^rd^ quartile; circles represent the outliers.
